# Association between vitamin D level in mother’s serum and the level of vitamin D in the serum of pre-term infants

**DOI:** 10.1186/s12887-023-03854-0

**Published:** 2023-03-02

**Authors:** Alireza Jashni Motlagh, Ahmadreza Davoodvandi, Sara Esmaelzadeh Saeieh

**Affiliations:** 1 Neonatal-Perinatal Medicine Department of Pediatrics, School of Medicine, Alborz University of Medical Sciences, Karaj, Iran; 2 Alborz University of Medical Sciences, Karaj, Iran; 3 Social Determinants of Health Research Center, Alborz University of Medical Sciences, Karaj, Iran

**Keywords:** Preterm, Vitamin D, Infant

## Abstract

**Background:**

Vitamin D deficiency and insufficiency is common in mothers and infants. The present study was conducted with the aim of association between the level of vitamin D in the mother’s serum and the level of vitamin D in the serum of preterm infants.

**Method:**

The present descriptive – analytical study was performed on 140 mothers and preterm infants referred to the Kamali hospital in Alborz University of Medical Sciences. Sampling was done from available mothers after hospitalization for delivery and their infants during the first 72 hours after delivery. Information from mothers and infants were collected with researcher made check list, including age, type of delivery, number of pregnancies, vitamin D during this pregnancy, infants birth age, gender, and birth weight. Data analysis was performed using SPSS version 24 software.

**Results:**

The average age of mothers was 28 ± 5 years and the average age of infants at the time of birth was 30 ± 1 weeks. Forty two infants (67%) were boys and 23 infants (33%) were girls. The results showed a high rate of vitamin D insufficiency and deficiency in mother (44, 49%) and preterm infants (49, 38%). The results of the Pearson correlation test showed that there is a strong and significant relationship between the serum vitamin D level of mothers and preterm infants (*P* = 0.001). Logistic regression tests showed that mother’s vitamin D level had an effect on the infant’s vitamin D level.

**Conclusion:**

Considering high rate of vitamin D deficiency and insufficiency and relationship between vitamin D in preterm infants serum and mothers, diagnostic screenings are recommended to investigate vitamin D disorders in pregnant mothers, which should be planned, implemented and followed up in the form of a therapeutic program to prevent the occurrence of complications caused by this disorder in the mother and infant.

## Background

Vitamin D has a very important role for the development of the fetus during the stages of cell proliferation, differentiation and maturation [1]. Optimal concentrations of vitamin D may influence early organogenesis and subsequently affect health status [2]. Also, vitamin D is critical for placental function, calcium homeostasis and bone mineralization, all of which are important factors for fetal growth and development [3]. During pregnancy, the serum level of vitamin D increases two times at 10-12 weeks pregnancy and reaches the maximum level in the third trimester [4]. However, due to the increase in the active form of vitamin D, pregnant women are probably exposed to more cells with vitamin D during the second and third trimesters, which suggests the role of vitamin D in the well-being of women [5]. In infancy, severe vitamin D deficiency with hypocalcemia can lead to convulsions in infants, and it has also been seen that low vitamin D concentration in umbilical cord blood has been associated with increased incidence of neonatal sepsis and respiratory tract infections in the first year of life [6]. Perinatal results suggest the hypothesis related to the role of vitamin D in the occurrence of preeclampsia, gestational diabetes, cesarean section, infectious diseases, low birth weight and preterm delivery [7, 8]. Preterm birth is the most important cause of death in children under 5 years of age [9]. It is estimated that 11% of all annual births in the world are preterm, and 1 million of every 6 million preterm newborns die from complications of prematurity. Twenty seven percent of all births in low- and middle-income countries are preterm for gestational age [10]. Preterm delivery is associated with bad health outcomes such as increased infant mortality, nutritional disorders, children’s vision and hearing, and metabolic diseases in adulthood [11]. Risk factors for preterm delivery include African-American race, poverty, young maternal age, obesity, and vitamin D deficiency. Vitamin D status in the fetus and newborn is largely determined by the vitamin D status of the mother [12]. According to the studies, the prevalence of vitamin D deficiency in the general population is very important, and pregnancy is one of the known risk factors for this deficiency, and in general, vitamin D deficiency has been reported between 47 and 83% in black and white pregnant women, respectively [13].

Since vitamin D deficiency is prevalent in mothers, many infants are also at risk of deficiency and insufficiency of vitamin D levels. Researchers have recently shown an adverse role of low vitamin D levels in health status beyond calcium metabolism and bone health, such as health status during pregnancy and in infancy and childhood [14]. Animal studies have shown that vitamin D may be important in lung development [15]. This factor is very important for preterm infants due to the high risk of chronic lung disease. The potential long-term public health impact of preventing vitamin D deficiency is being evident, especially at this critical time [16].

Since investigating the causes of preterm birth and identifying modifiable risk factors for this adverse birth outcome is necessary [17], in the present study, the relationship between vitamin D level in mother and preterm infants is investigated.

## Method

### Study design

The present descriptive – analytical study was performed after obtaining written permission from the Ethics Committee with the code IR.ABZUMS.REC.1399.235 on 140 mothers and pre-term infants referred to Kamali Medical Education Center in Alborz University of Medical Sciences. Sampling was done from mothers after hospitalization for delivery and from their infants during the first 72 hours after delivery. Serum levels of vitamin D in mother before delivery were measured by immunological and biochemical methods by sampling 2 ml of venous blood. In addition, the serum levels of vitamin D of infants were measured in the first 72 hours after delivery by immunological and biochemical methods by sampling 2 ml of venous blood. In this study, according to the guidelines of the International Endocrine Society, vitamin D deficiency was defined as a serum level less than 20 ng/ml, insufficiency was defined as 21-29 ng/ml, and its normal level was at least 30 ng/ml.

### Study population

Eligible criteria for entering the study were:

- Mothers with preterm infants less than 32 weeks;
- A healthy infant born with an age less than or equal to 32 weeks of pregnancy;

Exclusion criteria were:

- Parents’ unwillingness to cooperate, infant’s death immediately after birth, mothers with known underlying liver, kidney or heart diseases, taking drugs affecting calcium and vitamin D metabolism in the mother, chromosomal or congenital abnormalities of infants.

### Sample size

In this study, the sample size was determined based on Panda et al.’s study and the main goal was to determine the relationship between the serum vitamin D level of mothers and the serum vitamin D level of preterm infants [18]. The error level is 5% and the statistical power is 95%. Based on the sample size calculation formula to find correlation (according to the sample size calculation formula, the sample size is 15 preterm infants and 15 mothers) and also considering the minimum sample required to perform parametric analyzes of the minimum sample size, 30 mothers and 30 preterm infants were calculated. The sample size of this study was 70 mothers and 70 preterm infants.

The standard normal deviate for α = Zα = 1.960.

The standard normal deviate for β = Zβ = 0.842

$$\textrm{C}=0.5\ast \ln \left[\left(1+\textrm{r}\right)/\left(1-\textrm{r}\right)\right]=0.793$$

$$\mathrm{Total}\;\mathrm{sample}\;\mathrm{size}=\mathrm N=\;\lbrack(Z\mathrm\alpha+\mathrm{Z\beta})/\mathrm C\rbrack2\;+\;3\;=\;15$$

### Study questionnaire

The data collection tool in this study was a researcher made checklist to collect the demographic information of the mother and the infant and information on the time of delivery, including age, type of delivery, number of pregnancies, vitamin D and calcium supplementation during this pregnancy, and newborn information including birth age, gender, and birth weight. The content of this check list was confirmed by 10 faculty member of neonatology department.

### Sampling method

Sampling method was convenient, after the approval of the Vice-Chancellor of Research and the Ethics Committee of Alborz University of Medical Sciences, pregnant mothers with preterm delivery of less than 32 weeks were selected as available and after explaining the objectives of the study, the confidentiality of information were confirmed and informed consent was obtained.

### Data analysis method

The data was analyzed using SPSS version 24 software. The Kolmogorov–Smirnov test was used to determine the normal distribution of the data. Pearson correlation analysis was used to determine correlation and logistic regression was performed in order to determine the effect of demographic factors on vitamin D levels.

## Results

The average age of mothers was 28 ± 5 years and the minimum and maximum age was 18 and 37 years. The average age of infants at the time of their birth was 30 ± 1 week, and the minimum and maximum ages were 27 and 31 weeks. 47 infants (67.1%) were boys and 23 infants (32.9%) were girls. Regarding the use of vitamin D supplements during pregnancy, 6 mothers (9%) had taken vitamin D supplements. The level of vitamin D and calcium in mothers and infants is mentioned in Table 1. Also, the results of Pearson’s correlation test showed that there is a strong and significant relationship between the serum level of vitamin D of mothers and preterm infants (Table 1).

**Table 1:** Vitamin D level of mother and infant

Group	Vitamin D Status (ng/ml)	F(P)	*P*-Value*
Mother	Deficiency	34 (49%)	0.001 *r* = 0.79
	Insufficiency	31 (44%)	
	Sufficiency	5 (7%)	
	Total	70 (100%)	
Mean ± SD (VitD)	21 ±7		
Minimum )Maximum ((VitD)	7.1 (42.1)		
Infant	Deficiency	27 (38%)	
	Insufficiency	34 (49%)	
	Sufficiency	9 (13%)	
	Total	70 (100%)	
Mean ± SD (VitD)	22 ± 7		
Minimum )Maximum( (VitD)	5.7 (37.7)		

^*^Perason Test

Logistic regression was used to determine the effect of gender (boy), mother’s age, fetal age, mother’s vitamin D level, vitamin D supplement use during pregnancy and birth weight of infants on the probability of vitamin D level of more than 20 ng/ml in infants. Table 2 shows that the logistic regression model was significant only for the vitamin D level of mothers (*P* < 0.05).

**Table 2:** Logistic regression to determine the effect of non-dependent variables in predicting the probability of a level of more than 20 ng/ml of vitamin Din preterm infants

Response Variable	Class	Variable	β	S.E	*P* Value	OR
Mother Age	–	vitamin D more than 20 ng/ml	0.07	0.11	0.49	1.08
Infant Age	–	vitamin D more than 20 ng/ml	0.17	0.35	0.61	1.19
Infant Weight	–	vitamin D more than 20 ng/ml	−0.005	0.003	0.13	0.99
vitamin D Level of Mother	–	vitamin D more than 20 ng/ml	0.23	0.08	0.008	1.26
Gender	Boy	vitamin D more than 20 ng/ml	−1.58	1.12	0.15	0.204
vitamin D Complement	Consumption During Pregnancy	vitamin D more than 20 ng/ml	1.28	1.53	0.40	3.60

## Discussion

The findings from this study showed that 49% of mothers had vitamin D deficiency and 44% had vitamin D insufficiency. 38% of infants had vitamin D deficiency and 49% had vitamin D insufficiency. In Fallahi et al.’s study [13], the average level of vitamin D in infants was 13.7 ng and 56% of infants were vitamin D deficient.

Panda et al. [18] demonstrated that the level of vitamin D was 54.4 (36-70.7) n mol in mothers and 18.2% of mothers had vitamin D deficiency and 21.8% had insufficient vitamin D level. Also, the level of vitamin D at the time of birth of infants was 57 (42-70) and the prevalence of vitamin D deficiency in 72 days after delivery was reported as 30%.

Hajizadeh and et al. showed the high prevalence of hypovitaminosis D in the pregnant women of the Middle East underscores and they suggested the necessity of implementing national prevention and intervention strategies. A clear policy for clinicians and healthcare workers is needed for screening and maintaining sufficient vitamin D status during pregnancy [19].

In the present study, the Pearson correlation coefficient for the serum level of vitamin D in mothers and infants shows a strong and significant relationship between the serum level of vitamin D in mothers and preterm infants. In this context, Skouroliakou et al. reported a strong and significant correlation between the serum level of vitamin D in mothers and preterm infants, which confirms the results of the present study [20]. In the study of Mafinejad et al., a strong and significant relationship was demonstrated between serum levels of vitamin D in mothers and preterm infants, which is in line with the results of the present study. Similarly, a strong and significant relationship was also reported by Kassai et al. and Park et al. between serum levels of vitamin D in mothers and preterm infants [21–23]. Results of systematic review showed Maternal and newborn vitamin D level have correlation [24].

Results of another study showed that there is a strong mother/infant vitamin D relationship that affects vitamin D status during pregnancy and in infancy. Vitamin D deficiency is a universal health problem is a basis on which to improve guideline to reduce the outcome of maternal and infant vitamin D deficiency [25].

Logistic regression test was performed to determine the effect of gender (boy), mother’s age, fetal age, mother’s vitamin D level, vitamin D supplement use during pregnancy and birth weight of the on the infant’s vitamin D level, and the findings showed that only the mother’s vitamin D level more than 20 ng/ml affects the vitamin D level in infants.

Nakhaee and et al. showed that infants’ boy, low mothers’ vitamin D status, inadequate mothers’ receiving vitamin D supplementation, and high mothers’ parity are correlated with infant’s vitamin D status [26].

In this field, in the study of Skouroliakou et al., the logistic regression model showed the effect of mothers’ serum vitamin D level in predicting the level of more than 20 ng/ml of vitamin D in preterm infants, which confirms the results of the present study [20].

The results of the present study showed that the age of the mother is not significant for predicting the sufficient level of vitamin D in preterm infants, but in a study [27], higher levels of vitamin D in preterm infants of mothers over 30 years of age were reported. Considering the average age of mothers and their age range, this relationship may not be determined in the present study.

The results of the present study showed that the mean and frequency of vitamin D status of infants did not differ in terms of gender, which was also reported in the study of Skouroliakou et al. [20].

The present study demonstrated that the average birth weight of preterm infants in the present study was not statistically different according to the vitamin D status of the mothers. Because of the influence of various factors on birth weight, different results are reported. Wang et al. [28] showed the relationship between vitamin D and birth weight, so that for one nanogram increase in the amount of vitamin D, head circumference (0.31 cm) and the birth weight (69 g) are increased. Results of a trial study showed consumption maternal vitamin D supplement from two trimester of pregnancy until 6 months postpartum did not improve fetal or infant growth [29].

Kiely and et al. in a study showed that there were no associations between cord vitamin D and birth weight or any anthropometric measures at birth [30].

The results of the present study showed that there was no significant relationship between the consumption of vitamin D supplements by mother during pregnancy and the prediction of the normal level of vitamin D in preterm infants.

Roda (2015) in a clinical study reported that the use of vitamin D supplements by mothers in the intervention group increases vitamin D in the infant’s umbilical cord. The serum level of vitamin D in the infants of the intervention group was twice that of the control group [31].

Results of a study showed that Daily vitamin D supplementation during pregnancy and then infancy with 1000/400 IU or 2000/800 IU increases the proportion of infants with vitamin D ≥ 20 ng/mL, with the higher dose sustaining this increase for longer [32]. Hollis and et al. in a study showed that consumption of vitamin D supplementation of 4000 IU/d during pregnancy is safe and effective in level of vitamin D sufficiency in all women and their neonates [33, 34]. The present study was not consistent, which could be due to the fact that only 9% of the participants of the present study used vitamin D supplements. And also infant in this study was born preterm that might be effect the results of study.

Consumption of vitamin D supplementation during pregnancy has been studied since the past but decision making about vitamin D’s effects during pregnancy, dose and timing of supplementation are not clear [35].

Screening of vitamin D during pregnancy is an effective way in recognition of vitamin D deficiency and improving vitamin D levels [8].

The limitations of the present study include the limitation of the sample size and lack of examination of mothers’ diet, which plays a role in the prevalence of vitamin D deficiency. The second limitation was the lack of access to the mother’s vitamin D level at the beginning of pregnancy.

## Conclusion

The findings of the present study showed a high rate of insufficiency and deficiency of vitamin D in mothers and preterm infants. On the other hand, the serum level of vitamin D of the mother and the preterm infant had a strong and significant relationship, and the only predictor of the appropriate level of vitamin D in preterm infants was the level of vitamin D in the mother’s serum. Hence, diagnostic screenings are recommended to investigate vitamin D disorders in pregnant mothers, which should be planned, implemented and followed up in the form of a therapeutic and diagnostic program to prevent the occurrence of complications caused by this disorder in the mother and infant.

## Data Availability

The data that support the findings of this study are available from the corresponding author upon reasonable request.
